# Maternal hatching synchronization in a subsocial burrower bug mitigates the risk of future sibling cannibalism

**DOI:** 10.1002/ece3.3894

**Published:** 2018-02-22

**Authors:** Hiromi Mukai, Mantaro Hironaka, Sumio Tojo, Shintaro Nomakuchi

**Affiliations:** ^1^ Department of Forest Entomology Forestry and Forest Products Research Institute Tsukuba Ibaraki Japan; ^2^ Department of Biology Faculty of Medicine Hamamatsu University School of Medicine Hamamatsu Shizuoka Japan; ^3^ Department of Applied Biological Sciences Faculty of Agriculture Saga University Saga Saga Japan

**Keywords:** *Adomerus rotundus*, communal living, interclutch predation, maternal vibration, synchronous molting

## Abstract

Sibling cannibalism—the killing and consumption of conspecifics within broods—carries a high risk of direct and inclusive fitness loss for parents and offspring. We reported previously that a unique vibrational behavior shown by the mother of the subsocial burrower bug, *Adomerus rotundus* (Heteroptera: Cydnidae), induced synchronous hatching. Maternal regulation may be one of the most effective mechanisms for preventing or limiting sibling cannibalism. Here, we tested the hypothesis that synchronous hatching induced by maternal vibration in *A. rotundus* prevents sibling cannibalism. Mothers and their mature egg masses were allocated to three groups: synchronous hatching by maternal vibration (SHmv), synchronous hatching by artificial vibration (SHav), and asynchronous hatching (AH). We then investigated the influence of each hatching strategy on the occurrence of sibling cannibalism of eggs and early‐instar nymphs in the laboratory. No difference in the proportion of eggs cannibalized was observed among the three groups. However, the proportion of nymphs cannibalized was higher in the AH group than in the SHmv group. The difference in the number of days to first molting within clutch was significantly higher in the AH group than in the SHmv group. Junior nymphs were sometimes eaten by senior nymphs. However, immediately after molting, senior nymphs were at a high risk of being eaten by junior nymphs. Our results indicate that synchronous hatching of *A*. *rotundus* is necessary to mitigate the risk of sibling cannibalism.

## 1 INTRODUCTION

Cannibalism is observed in many animal taxa exhibiting various feeding habits and not only in predators but also in herbivores (Elgar & Crespi, 1992; Polis, 1981). Sibling cannibalism is common in both vertebrates (Fitzgerald & Whoriskey, 1992; Kam, Lin, Lin, & Tsal, 1998; Stanback & König, 1992) and invertebrates (Baur, 1992, 1993; Iida, 2003; Kudo & Nakahira, 2004; Liebig, Monnin, & Turillazzi, 2005; Osawa, 2002; Schausberger, 2004, 2007) with offspring that are aggregated in nests.

Sibling cannibalism is adaptive in some ecological backgrounds, as it may be an important means of obtaining a rich nutrient supply for the survival and development of offspring. Cannibalism may benefit parents if they ultimately gain more offspring under starvation conditions because of enhanced survival of the cannibals compared with the victims lost (Crespi, 1992). Cannibalism in many insects is directed toward siblings in the defenseless egg stages (e.g., Branquart, Hemptinne, Bauffe, & Benfekih, 1997; Sigsgaard, Greenstone, & Duffield, 2002; Via, 1999). Therefore, parents have developed strategies to decrease hatching synchrony in low‐food environments to facilitate cannibalism among their offspring (Forbes, Grosshans, & Glassey, 2002; O'Connor, 1978). However, sibling cannibalism usually carries a high risk of direct fitness loss for the mother and inclusive fitness loss for her offspring (Hamilton, 1964a, 1964b). Adaptive mechanisms that prevent or limit sibling cannibalism may evolve under these circumstances, although to our knowledge, little effort has been made to examine these mechanisms.

**Figure 1:**
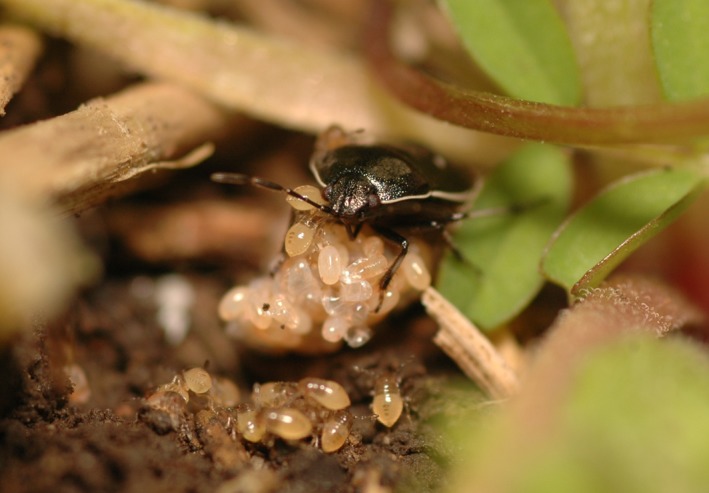
The study species; a subsocial burrower bug, *Adomerus rotundus*. Mothers produce physical vibrations that regulate and synchronize hatching. Picture: HM and MH

Hatching is extremely well synchronized in some species of burrower bugs and closely related shield bugs (Mukai, Hironaka, Tojo, & Nomakuchi, 2012, 2014). These species show complex maternal care, including egg guarding, production of trophic eggs, protection of nymphs, and progressive provisioning (Baba et al., 2011; Filippi, Hironaka, & Nomakuchi, 2001; Filippi et al., 2009; Hironaka, Nomakuchi, Iwakuma, & Filippi, 2005; Inadomi et al., 2014; Mukai et al., 2010; Nakahira, 1994; Tsukamoto & Tojo, 1992). We reported previously that, while holding their egg masses, *Adomerus rotundus* (Heteroptera: Cydnidae) mothers produce physical vibrations that regulate and synchronize hatching (Figure 1; Mukai et al., 2012). This sophisticated care behavior probably has the adaptive function of supporting the young offspring. *A. rotundus* feeds mostly on seeds of the host plant *Lamium amplexicaule* (Lamiales: Lamiaceae), but nymphs occasionally kill and feed on conspecifics. We hypothesized that maternal synchronization of hatching would function to prevent cannibalism among siblings. To test our hypothesis, we prepared unsynchronized and naturally or artificially synchronized hatching broods and investigated the effects on the occurrence of sibling cannibalism during early development in the laboratory.

## 2 MATERIALS AND METHODS

### 2.1 Experimental animals

*Adomerus rotundus* oviposits a spherical egg mass on the ground and guards the eggs for 6 or 7 days until hatching (Inadomi et al., 2014; Mukai et al., 2012). After these eggs hatch, the mothers produce trophic eggs and give them to the hatchlings as their first food, and then frequently leave the nest to collect seeds of the host plant (Inadomi et al., 2014). This maternal care usually lasts until the late second instar or early third instar—approximately 7 days.

The study insects came from a stock population originating from adults collected in Chinzei‐machi, Karatsu, Saga, Japan, between late April and early May 2008. This population is maintained in a laboratory in the Department of Agriculture at Saga University. In total, 100 prereproductive adults were placed in a plastic container (14 × 20 cm and 6 cm high) containing moist laboratory wipes and maintained in an incubator at 27°C under 16 hr light: 8 hr dark. We gave the adults sufficient host plant seeds every day.

### 2.2 Experimental procedures

Seventy mothers and their mature egg masses were allocated to three groups: synchronous hatching by maternal vibration (SHmv), synchronous hatching by artificial vibration (SHav), and asynchronous hatching (AH). Females in SHmv were left with their egg masses at the moment of hatching, and all egg masses hatched within 15 min (*N *=* *28). These females were then removed after the hatching. Females in SHav were moved away from their egg masses 24 hr before the expected hatching, and artificial vibration was provided by an electrodynamic cordless motor (Pellet Pestle Cordless Motor; Kimble Chase Life Sciences and Research Products LLC, Vineland, NJ, USA). We attached a motor one by one in contact with a glass dish, in which an egg mass was placed, and turned the switch on and off on an average of 30 times/min during the first 10 min and then 10 times/min for 11–20 min to simulate the maternal vibration pattern (Mukai et al., 2012). All egg masses hatched synchronously within 15 min with this method (*N *=* *11). AH females were moved away from their egg masses 24 hr before the expected hatching. In this group, the difference in hatching time between first nymph emergence and last nymph emergence in a brood was more than 3 hr (*N *=* *31). We used egg masses consisting of 50–60 eggs for all three treatments.

To evaluate only the influence of synchrony of hatching, and not feeding conditions, on cannibalism, all hatchlings were starved during the treatments. We used forceps to remove all trophic eggs from the egg mass surface. Viable eggs developed pinkish embryos with pigmented eyespots and were easily distinguished from nonviable trophic eggs. Six days after oviposition, we transferred the egg masses to clear plastic dishes (4 cm diameter, 1 cm height), containing moist absorbent cotton. Each group was kept in an incubator at 27°C under 16 hr light: 8 hr dark and checked daily at 10:00 a.m. All egg masses were observed under a microscope 24 hr after hatching. We counted the number of hatchlings and calculated the percentage of embryos that had, or had not, been cannibalized.

We then observed nymphal cannibalism and differences in the number of days to molting within each clutch during communal living. We assigned females and nymphs from the initial part of the experiment to the same experimental groups after hatching. Thirty‐two mothers and their broods were reassigned to the three groups 24 hr after hatching: SHmv (*N *=* *11), SHav (*N *=* *11), and AH (*N *=* *10). We chose 30 nymphs with no injuries or locomotor abnormalities from each brood and transferred them to a clear plastic dish (4 cm diameter, 1 cm height) containing moist absorbent cotton. We provided two host plant seeds per dish and checked the nymphs daily at 10:00 a.m. We counted the number of cannibalized nymphs and the percentage of nymphs that had, or had not, been cannibalized. Nymphs with bodies that had collapsed from sucking by a sibling were defined as cannibalized nymphs. We checked the numbers of nymphs in each stage daily among the groups and calculated the maximum difference in the number of days to first molting within each clutch (from first emergence of second‐instar nymphs to disappearance of first‐instar nymphs in a brood). The experiment was stopped 7 days after hatching; nymphs usually grow from the first to the third instar during this period. The first instar is from 0 to 4 days after hatching, the second is from 3 to 7 days after hatching, and the third appears after 7 days, by which time, under natural conditions, almost all of the nymphs would have moved away from their mothers. Nymphs younger than the eaten individuals were categorized as “junior nymphs,” and nymphs older than the eaten individuals were categorized as “senior nymphs” (Sainte‐Marie & Lafrance, 2002; Schausberger & Hoffmann, 2008).

### 2.3 Statistical analyses

Statistical analyses were conducted with R version 3.2.1 software (R Development Core Team, 2015). Cannibalism rates and egg viability were analyzed using a Kruskal–Wallis test, and multiple comparisons were performed with the Steel–Dwass test. The number of days to first molting was analyzed using a one‐way ANOVA, and multiple comparisons were made with Tukey's HSD test. The correlation between the occurrence of sibling cannibalism and the maximum within‐clutch difference in the number of days to first molting was analyzed using the least‐squares method.

## 3 RESULTS

**Figure 2:**
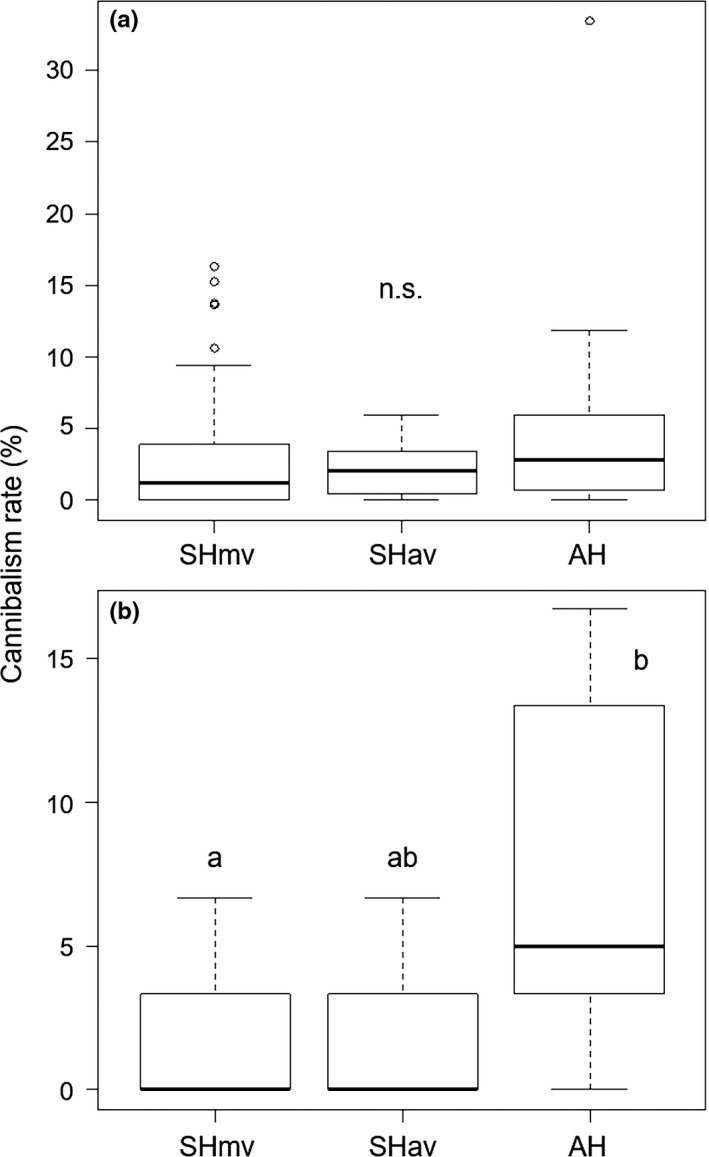
Box plots showing differences in cannibalism rates (a) 24 hr after detection of hatching (embryo–nymph interaction) and (b) 24 hr to 7 days after hatching (nymph–nymph interaction) among SHmv, SHav, and AH groups. In the box plots, horizontal line within the box is the median; box indicates the lower and upper quartiles; capped vertical lines are 95% confidence limits, and white dots are outliers. Different letters indicate significant differences (Steel–Dwass test, *p *<* *.05). SHmv, synchronous hatching by maternal vibration; SHav, synchronous hatching by artificial vibration; AH, asynchronous hatching

Egg viability did not differ across treatments (Figure S1; Kruskal–Wallis test, $X^{2}$ = 3.32, *df* = 2, *p *=* *.20). Rates of egg cannibalism did not differ significantly among the three groups (Figure 2a; Kruskal–Wallis test, $X^{2}$ = 1.69, *df* = 2, *p *=* *.43). However, the rate of nymph cannibalism differed significantly (Figure 2b; Kruskal–Wallis test; $X^{2}$ = 6.78, *df* = 2, *p *=* *.03); according to the Steel–Dwass test, the rate was again significantly higher in AH than in SHmv (*p *=* *.03), and it was marginally higher than in SHav (*p *=* *.06).

**Figure 3:**
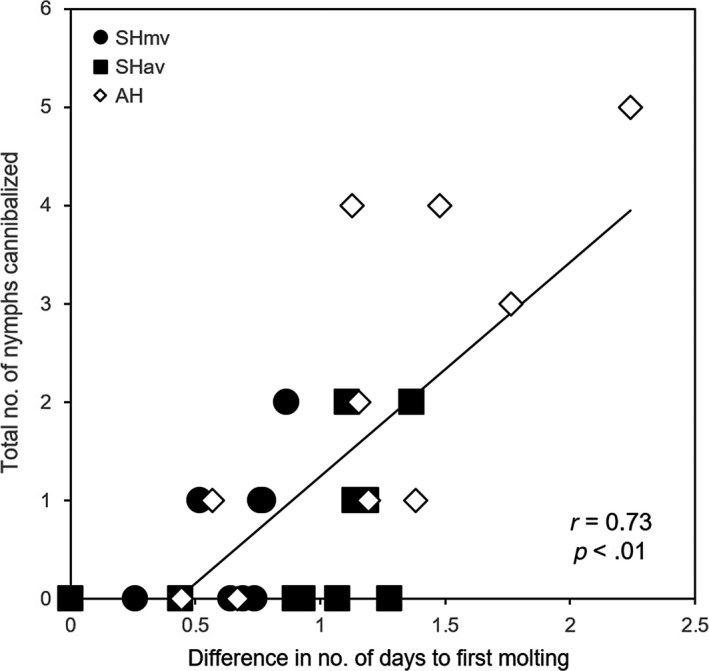
Relationships between differences in the number of days to first molting and total number of nymphs cannibalized. SHmv, synchronous hatching by maternal vibration; SHav, synchronous hatching by artificial vibration; AH, asynchronous hatching

**Figure 4:**
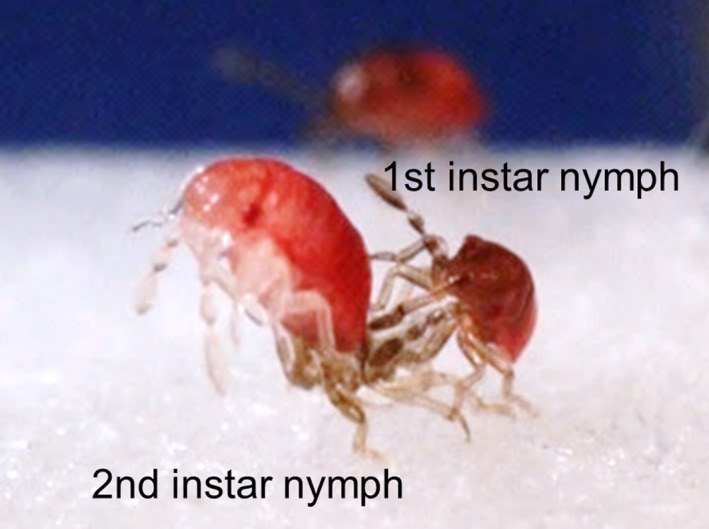
Sibling cannibalism of newly molted second instar by first instar

**Figure 5:**
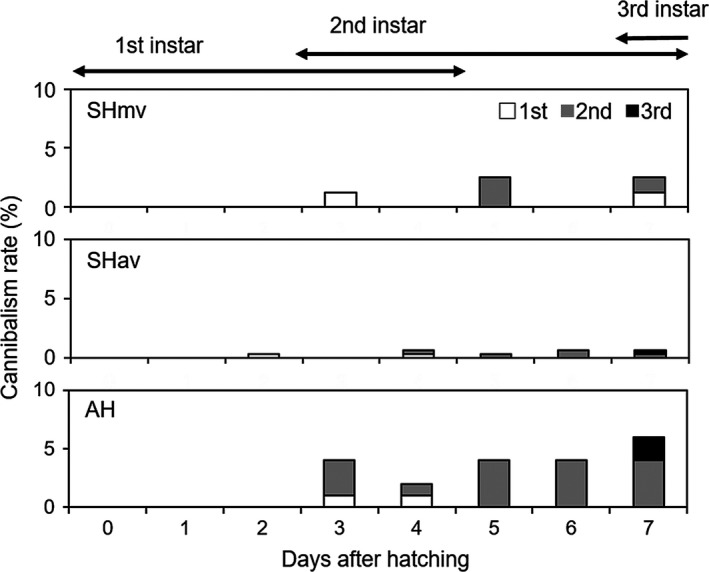
Timing of sibling cannibalism in the same broods. Duration of instars is indicated above the graph. White columns, cannibalized first‐instar nymphs; gray columns, cannibalized second instars; and black columns, cannibalized third instars. SHmv, synchronous hatching by maternal vibration; SHav, synchronous hatching by artificial vibration; AH, asynchronous hatching

Nymphal molting was observed from 3 days after hatching. Before molting, nymphs tend to remain sedentary for a few days as they prepare for the next molt. They then shed the exuvium over several hours. The difference in the number of days to first molting within clutch differed significantly among the three groups (Figure 3; one‐way ANOVA, *F*_2, 29_ = 5.04, *p *=* *.01): In AH, it was significantly higher than in SHmv (Tukey's HSD test; *p *=* *.01). There was a positive relationship between the number of nymphs cannibalized and the maximum difference in the number of days to first molting in all groups (Figure 3; *r *=* *.73, *p *<* *.0001). Approximately half the AH clutches took longer to reach the first molt and showed higher cannibalism rates than the SHmv and SHav clutches (Figure 3). In AH, we frequently observed nymphal cannibalism across siblings within a brood. Occasionally, a few first‐instar nymphs were observed inserting their proboscises into newly molted second‐instar nymphs (Figure 4). Rates of nymphal cannibalism especially in AH increased from 3 days after hatching, when the first molts started (Figure 5).

## 4 DISCUSSION

We found that the absence of maternal or artificial vibration facilitated AH of egg mass and induced asynchronous molting, which increased the rate of cannibalism between junior and senior nymphs. These results support our hypothesis and provide experimental evidence that maternally regulated synchronous hatching mitigates the risk of sibling cannibalism (Fréchette & Coderre, 2000).

Sibling cannibalism is most intense during the early nymphal stages in many animals, when embryos and nymphs exist in a community group because the first meal after hatching is particularly important for growth, survival, and overall performance later in life (Polis, 1981). It is traditionally thought that senior nymphs eat junior nymphs. However, our findings in *A. rotundus* revealed that senior nymphs are not always predators. Senior nymphs never ate eggs that had not yet hatched, but they occasionally ate junior nymphs at the molting phase. However, starving junior nymphs cannibalized senior nymphs that had just molted (Figures 4 and 5). Such within‐brood interactions caused by AH may have been an evolutionary driving force for the unique maternal hatching regulatory system and synchronous hatching in *A. rotundus*.

Many ethologists and ecologists have tried to clarify the conditions that facilitate and prevent sibling cannibalism. Previous studies of sibling cannibalism have reported that parents are likely to promote sibling rivalry and cannibalism when their offspring have a poor survival chance (Elgar & Crespi, 1992; Perry & Roitberg, 2006). Small and weak junior offspring are eaten by senior siblings when food is scarce to increase the survival chance of the senior siblings (Forbes et al., 2002; Mock & Forbes, 1995). Parents in some bird and insect species appear to increase hatching asynchrony to facilitate sibling cannibalism (Wiebe & Bortolotti, 1994; Fréchette & Coderre, 2000; but see Schausberger & Hoffmann, 2008). Thus, changes in hatching synchrony within a brood might affect the occurrence of sibling cannibalism: AH facilitates, whereas synchronous hatching limits, sibling cannibalism.

Studies in Coleopteran species reported that hatching synchrony is unrelated to sibling egg cannibalism (Kutcherov, 2015; Perry & Roitberg, 2005). In *A. rotundus*, we found no differences among the three groups in the percentage of eggs cannibalized (Figure 2a). It might be difficult for hatchlings to insert their proboscises into viable eggs, which are covered by a hard shell. Thus, the eggs may be protected physically and possibly chemically. On the other hand, the percentage of nymphs cannibalized in AH was significantly higher than those in SHmv (Figure 2b). Senior nymphs develop and molt faster than junior nymphs because they can monopolize food resources such as trophic eggs and the plant seeds provided by the parents under natural conditions. Even though early development seems adaptive, in fact, senior nymphs that have just molted are occasionally eaten by junior nymphs. Molting individuals are usually vulnerable, because they have a thin integument and cannot escape or protect themselves from conspecific or interspecific predation (Dick, Irvine, & Elwood, 1990; Soluk, 1990). In some communal‐living spiders, extreme developmental synchrony reduces sibling cannibalism (Johnson, Halpin, & Stevens, 2016). It is crucially important that we focus not only on the eggs but also on all life stages in examining the effects of hatching synchrony in gregarious species.

Trophic eggs are thought to be laid as meals for hatchlings (Alexander, 1974; Crespi, 1992), but changes in their production are strongly associated with changes in rates of cannibalism (Perry & Roitberg, 2006). In the Asian ladybird, *Harmonia axyridis* (Coleoptera: Coccinellidae), mothers manipulate the production of trophic eggs under starvation conditions to ensure cannibalism and thus reduce the numbers of their offspring (Perry & Roitberg, 2005). As is the case in the closely related species *Adomerus triguttulus*, it is thought that the trophic eggs of *A. rotundus* function only to support hatchling nutrition and have no other special function (Baba et al., 2011; Kudo & Nakahira, 2004). Here, we removed all trophic eggs to standardize the nutritional conditions. In fact, it was impossible to standardize the amount of feeding on trophic eggs across treatments, because nymphs will eat trophic eggs that have just been laid by the mothers. *A. rotundus* mothers intermittently produce just over a dozen trophic eggs as late as before and after the fertilized eggs have begun to hatch (Inadomi et al., 2014). In SHav and AH, the mothers were completely removed before hatching started; therefore, under the experimental condition, it is expected that the hatchlings in these groups are unable to eat any trophic eggs laid after they have hatched. Because of the constraints of the treatment conditions, it was practically impossible to test for effect of hatching synchrony on cannibalism in the presence of a specific number of trophic eggs. However, in our preliminary experiment, SHmv groups given trophic eggs both pre‐ and posthatch had egg and nymph cannibalism rates of about 1.0%; this is clearly lower than those of SHmv, SHav, and AH in our current experiment, which had no trophic eggs (Mukai, 2014). These findings suggest that trophic eggs play an extremely important role in mitigating cannibalism in *A. rotundus*.

## CONFLICT OF INTEREST

None declared.

## AUTHOR CONTRIBUTION

HM, MH, and ST conceived and designed the experiments. HM and MH performed the experiments. HM and SN analyzed the data. HM, MH, and SN wrote the manuscript.
