# Supplementary figures and images for: Maternal hatching synchronization in a subsocial burrower bug mitigates the risk of future sibling cannibalism
Source: Ecol Evol. 2018 Feb 22;8(6):3376–81. doi: 10.1002/ece3.3894 (PMC5869296; doi:10.1002/ece3.3894)

## Slide 1
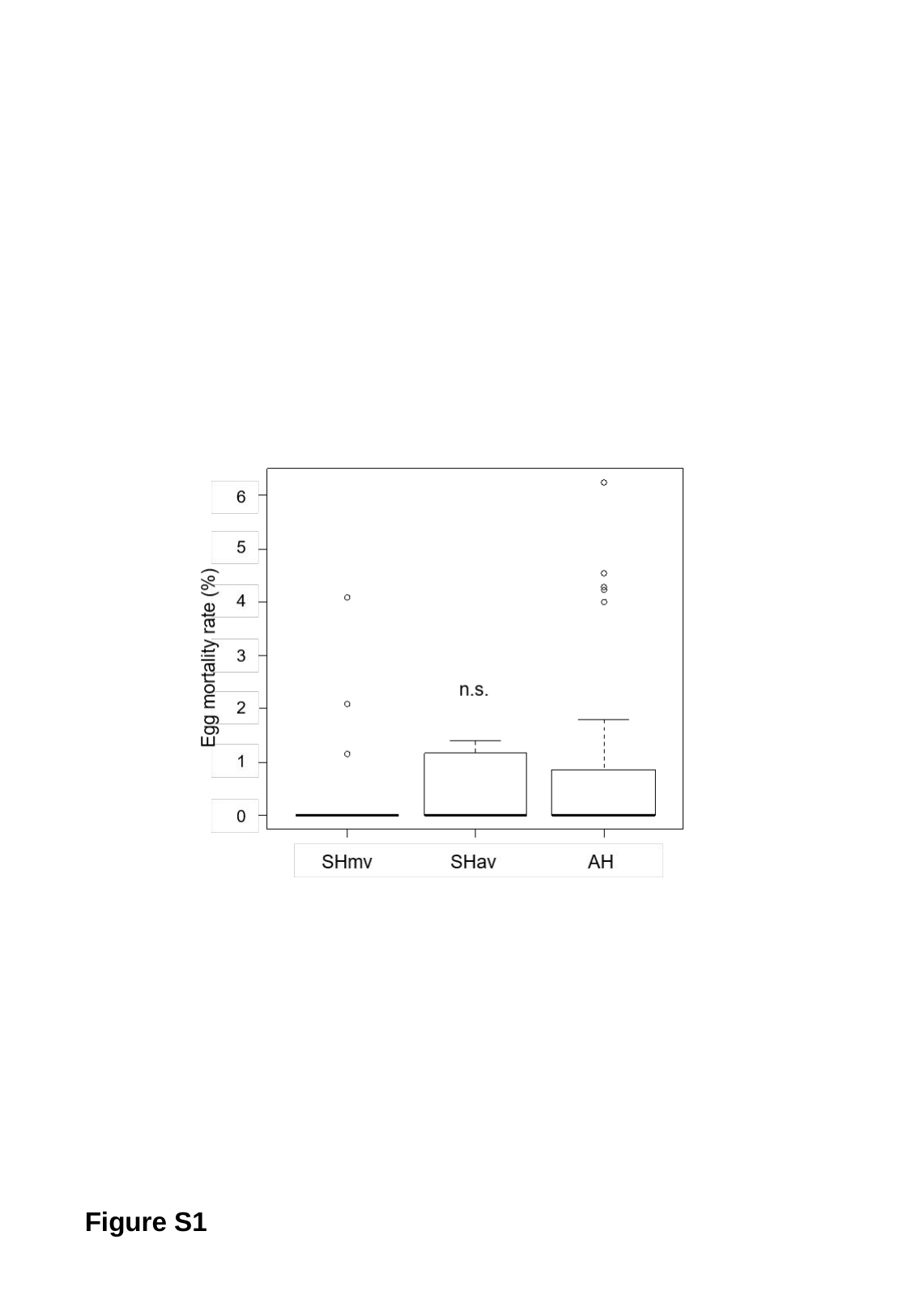

Figure S1

Supplement: Supplementary file 1 [file ECE3-8-3376-s001.pptx]
